# Mr. Hare, on Apoplexy

**Published:** 1802-05

**Authors:** Lancelot Hare

**Affiliations:** Member of the Royal College of Surgeons, London. Southminster, Essex


### *To the Editors of the Medical and Phyfical Journal.*

Gentlemen,

It is much to be lamented, that of all the liberal profeffions the conduct of the members of the Medical Profeffion to each other is, perhaps, the moft illiberal; and that this often arifes from an improper interference in each other's department, I have no doubt. Surely, Phyficians would act a more dignified and politic part, not to affume the functions of the Surgeon, Apothecary, or Nurfe; nor the Apothecary to ape the Phyfician; each confining himfelf to his proper fphere, would avoid much fchifm among its members.

The Controverfy upon Apoplexy between Dr. Langflow and Mr. Crowfoot, led me to thefe reflections. It would appear, you hefitated before you admitted it into your Journal, as being of too perfonal a nature. Had you rejected it, the lofs to the medical world would have been, in my opinion, very great, and in a point of view not connected with Apoplexy, nor noticed by any of your Correfpondents; I mean, as affording the moft moſt perfect example of its kind perhaps on record, to the Medical Profeſſion, how they ought to conduct themſelves to each other, by copying the moderation of the Phyſician, and avoiding the petulance of the Apothecary.

As I differ in ſome important points of theory and practice from both thoſe Champions, I may, without preſumption, be allowed to enter the liſts. I would ſet out with obſerving, that the queſtion reſpecting the propriety or impropriety of adminiſtering Emetics in Apoplexy, has been taken up too generally; there are caſes occurring daily, where they may be adminiſtered with propriety, and others in which it would be highly improper; therefore, no certain rule can, nor general rule ought, to be laid down. It is in the diſcrimination of diſeaſes, and the application of general principles to particular caſes, that one man's practice ſo much excels another's.

I believe it is as generally an admitted axiom as any in Phyſic, that in all caſes of true Apoplexy and Hemiplegy, there is extravaſation or effuſion of blood or lymph upon the brain. This was the opinion of that great luminary Mr. John Hunter, whoſe authority no man of ſcience will be diſpoſed to doubt. Taking this then for granted, I believe all theory is againſt the adminiſtration of Emetics, and all practical experience, under certain circumſtances, for them.

The circumſtances I refer to are, where patients have an attack after a full meal; I contend, *that after topical bleeding* the ſpeedieſt mode of relief is evacuating the ſtomach by an emetic, or exciting vomiting by tickling the throat with a feather.

It has been triumphantly aſked by Dr. Langſlow, how a *foul* ſtomach, and by others, how the ſtomach could be any cauſe of Apoplexy? Or how Dr. Fothergill could adviſe Emetics, ſo contrary to all reaſon and true ſcience? All this is eaſily anſwered and explained, viz. from the daily occurrence of this diſeaſe almoſt immediately after a full meal, as lately happened to the Archbiſhop of Milan, at the famous Conſulta at Lyons. Now, admitting this, it will naturally follow, that the ſtate of the ſtomach is a very frequent cauſe of Apoplexy; and I conceive, the mode in which it acts is chiefly by its diſtenſion, thereby impeding the free return of the blood from the head: Not from any *foulneſs* of ſtomach, nor quality of food, but merely from quantity. I myſelf almoſt daily experience the apoplectic effect of a full meal, after dinner, by an almoſt irreſiſtible propenſity to ſleep. Now, that there is a particular determination of blood to the head, at thoſe times, and that the ſtomach is the cauſe of that, no man can reaſonably doubt. I think it probable, nervous ſympathy may be conjoined with diſtenſion of ſtomach to produce this effect: Therefore, I conceive ceive that evacuating the ſtomach by vomitting, after topical bleeding, will take off the preſſure, by giving more room, admit the free return of the blood from the head, and be attended with leſs danger, than waiting for the more ſlow mode of evacuations by the inteſtines, from theoretical ideas of the hazard of determining the blood to the head by Emetics. In other words, that the certain relief often produced by them, more than counterbalances any ſuppoſed danger from their adminiſtration.

It has been petulantly aſked, if the cauſe of the diſeaſe be in the ſtomach, whence ariſes the inſtant relief often procured by bleeding? I would anſwer this query, by referring to a fact well known to farmers, viz. that they loſe many valuable cows annually, by turning them into freſh clover, or other pecculent food; this cauſes violent diſtenſion of the ſtomach and bowels by the extrication of the fixed air, and is by them termed *being blown*; the common idea is, that they burſt before death; I conceive that to be erroneous, and more probable, that they die apoplectic. The uſual mode of relief, in this part of the country, is to ſtab them with a knife, in the flank, in order to evacuate the air. Some years ago I had a fine cow *blown*, and thought it a better mode to introduce a trocar in the flank, as the canula could eaſily be kept open, and in the other way the lips of the wound ſoon cloſe; it had the deſired effect, but ſhe recovered from the effects of it very ſlowly. I mentioned the circumſtance to a practical farmer of the firſt information; he aſſured me, if I had had her bled, it would have anſwered better, and that he had ſaved ſix of his own cows one morning by bleeding them inſtantly; of the others, ſome were dead, and dying, before he reached them.

Dr. Moſsman, page 206 of your laſt Journal, ſays, "The "ſchool diviſion of this diſeaſe into ſanguineous and ſerous, "is very generally and juſtly exploded, and were it true would "admit of no beneficial application in practice." This I beg leave to deny in toto; that there may be, and is, lymphatic plethora, as well as venal or arterial, every day's experience convinces me, and that the treatment ought to be different I am well ſatisfied. I have now three caſes of Hemiplegy, that commenced with apoplectic attacks; they are all far advanced in life, and from their pale, ſallow complexions, you would conclude there was not an ounce of blood in the whole ſyſtem: Surely, they require a different mode of treatment, and the diſeaſe aroſe from a different cauſe than ſanguineous plethora. The mode of relief that I purſued with moſt ſucceſs has been to adminiſter briſk cathartics in the firſt inſtance, then the ſtimulating and diuretic plan, ſucceeded by tonics. The topical application of bliſters to the whole head head, in both ſpecies of Apoplexy, ought not I think to be diſpenſed with. If ever the ſcience of Anatomy ſhould arrive at that degree of perfection, to enable us to open lymphatics as we do veins or arteries, that would be the moſt ſcientific mode of relief.

Dr. Langſlow dwells much on ſerous or lymphatice *exudation*, as a cauſe of Apoplexy; I conceive exudation can never cauſe Apoplexy, and that both ſpecies is always cauſed by *rupture* of veſſels; becauſe it is the *ſuddeneſs* of the extravaſation that deſtroys the functions of the brain. In caſes of exudation it is productive of a different diſeaſe, hydrocephalus, and often the accumulation of lymph is ten times greater than in Apoplexy before it cauſes death, on account of its *gradual* production. Thoſe obſervations will apply to Dr. Langſlow's reaſoning upon his ſuppoſed caſe of Apoplexy in your laſt Number. I admired his ſplendid diſplay of fine writing at the outſet of this Controverſy; but it gives me pain to add, that I think there is a great *falling off* in matter and manner, in your laſt Journal, page 273.

It muſt ſtrike every Practitioner, that *general* blood-letting is carried to too great an extent; I refer particularly to a caſe of Mr. Wilkinſon's, page 144, where he took a large baſon full of blood, which evidently laid the foundation of his death, by bringing on dropſy. Likewiſe one of Mr. Davies, page 76, which evidently haſtened the patient's death. It would ſurely be a ſafer mode of practice, in all caſes of Apoplexy, to bleed as near the ſeat of the diſeaſe as poſſible, viz. by cupping, opening the temporal artery, or the jugular vein. In caſes of ſuch urgency, I think the application of leeches, in the firſt inſtance, too ſlow a mode of relief.

Without making any perſonal applications, it muſt be obvious, that many of your Correſpondents are too fond of *ſplitting* Apoplexies into nervous, puerperal, &c. In ſhort, if ſuch a mode of diviſion be encouraged, I ſhould not wonder to ſee every caſe of ſudden death attributed to Apoplexy. It reminds me of a celebrated Divine, in the laſt century, who undertook to explain the miracle of our Saviour's caſting a *legion* of devils out of a man poſſeſſed, which entered into a *herd* of ſwine. His firſt care was to aſcertain the number of men a legion conſiſted of; he found it varied at different times of the Roman Republic; he took the mean number: His next ſtudy was to find the number of ſwine in a herd; he then calculated the proportion of devils to ſwine, and found that there were about two devils and a half, or two devils and three-quarters, to each ſwine. He was ever after called Dr. *Split-Devil.*

LANCELOT HARE,

Member of the Royal College of Surgeons, London.

